# Fine-scale temporal and spatial dynamics of *Ae. albopictus* response to larviciding with *Bacillus thuringiensis israelensis* in Heidelberg, Germany

**DOI:** 10.1038/s41598-026-46094-9

**Published:** 2026-04-08

**Authors:** Charles R. S. Hatfield, Pascale C. Stiles, Prasad Liyanage, Jerome N. Baron, Artin Tokatlian Rodriguez, Norbert Becker, Alexander Zipf, Michael Beigl, Joacim Rocklöv

**Affiliations:** 1 Interdisciplinary Centre for Scientific Computing, Heidelberg University, Heidelberg, Germany; 2 TECO, Karlsruhe Institute of Technology, Karlsruhe, Germany; 3 Heidelberg Institute for Geoinformation Technology, Heidelberg, Germany; 4 Heidelberg Institute of Global Health, Heidelberg University Hospital, Heidelberg, Germany; 5 ICYBAC Mosquito Control GmbH, Speyer, Germany; 6 GIScience Research Group, Heidelberg University, Heidelberg, Germany; 7 Department of Epidemiology and Global Health, Umeå University, Umeå, Sweden

**Keywords:** Diseases, Ecology, Ecology, Zoology

## Abstract

The mosquito *Aedes albopictus* is expanding across Europe, raising concerns due to its ability to transmit arboviruses. The larvicide *Bacillus thuringiensis subsp. israelensis* (Bti) plays a central role in controlling the spread of *Ae. albopictus*. The objective of this study is to assess the temporal effectiveness of Bti as a control method using fine-scale spatiotemporal data and a distributed lag non-linear modelling framework. We analyzed 1,320 ovitrap observations from 195 traps alongside records of 4,387 Bti treatments and local environmental conditions for the entire 2023 mosquito season in Heidelberg, Germany. Bti treatments produced a clear reduction in egg counts, with the strongest marginal effects occurring 6–13 days after treatment with efficacy diminishing thereafter at lower treatment counts, while higher counts demonstrated sustained effects. Cumulatively, Bti was highly effective at reducing egg counts even at moderate treatment levels, and a counterfactual no Bti scenario suggested that Bti treatments reduced seasonal egg production by an estimated 41.9% (95% CI 24.4% – 58.5%) and prevented total establishment across the study site. These results demonstrate that Bti can substantially reduce *Ae. albopictus* populations in urban settings, though its limited temporal efficacy underscores the need for repeated interventions to prevent establishment and spread.

**Supplementary Information:**

The online version contains supplementary material available at 10.1038/s41598-026-46094-9.

## Introduction

*Ae. albopictus*, an invasive mosquito species originally native to Southeast Asia, is rapidly expanding its range across Europe, facilitated by rising temperatures due to anthropogenic climate change as well as increased international trade and human mobility.^1–4^ In Germany, the species has steadily progressed northward from the Swiss border and has established populations in over 40 districts as of 2024, including populations in Berlin, Munich, Bonn, and the neighboring areas of Frankfurt.^5^ Its continued spread poses a growing public health concern, as *Ae. albopictus* is a competent vector for several arboviruses, including Chikungunya and Dengue.^2^ In countries such as France and Italy, this threat has already materialized in the form of locally acquired Dengue outbreaks, which are increasing in both frequency and severity due to the establishment of substantial *Ae. albopictus* populations.^6,7^ Additionally, 2025 saw unprecedented Chikungunya outbreaks with 755 cases across 76 clusters in France, one third of which remain active, and 268 cases in Italy with 755 and 268 cases as of October 2025^8^ with cases recorded close to the German border. With the warming in Europe being twice as fast as the global pace, the impact of climate change is already leading to increased transmission suitability, widened transmission season and geographical expansion of transmission risk.^9,10^.

To contain the spread of *Ae. albopictus* and to limit the risk of outbreaks, public health authorities rely on a range of vector control measures, among which the naturally occurring bacterium—*Bacillus thuringiensis subsp. israelensis* (Bti)—plays a central role for larviciding.^11^ Bti has been used for several decades and is widely regarded as effective in both laboratory and field studies^12^. However, key questions remain regarding its operational efficacy, particularly in urban environments, where the common presence of cryptic breeding sites challenge treatment coverage.^13–16^ In particular, though many laboratory studies and commercial Bti products state an efficacy period up to 30 days, field testing has raised questions regarding the true efficacy of Bti across the full 30 day period.^17^.

Previous research has established variable efficacy of Bti among mosquito species, including for *Ae. albopictus*.^16^ However, as Europe grows to embrace greater levels of mosquito control to combat the proliferation of *Ae. albopictus,* it is imperative that we establish an understanding of how the efficacy of Bti varies across time. Without such knowledge it challenges efforts to design robust and efficient control regimes that do not overly engage the public, thereby risking engagement fatigue. This is important in areas where the public may not be used to mosquito control and thus may be hesitant to support large-scale and frequent vector control. In such situations, to manage popular support for mosquito control, we must be able to establish effective control regimes that balance effective control with public engagement.

To address these knowledge gaps, we evaluate the field efficacy of Bti as a larval source control in Heidelberg, Germany, by applying a distributed lag non-linear model (DLNM) framework to study the non-linear and lagged effects of Bti applications and weather on *Ae. albopictus* egg abundance. Specifically, using spatiotemporal data on Bti applications, mosquito egg counts, and environmental variables, we estimate the persistence of Bti efficacy over time and space and assess its interaction with microclimatic conditions and urban green spaces. A DLNM framework was selected given that Bti is a larvicide, which targets immature mosquitoes rather than adult ovipositing females. As a result, reductions in egg counts due to Bti occur indirectly and with a delay corresponding to the time it would take for mosquito larvae to develop into adults and reproduce. A DLNM approach therefore allows us to capture a delayed and potentially non-linear relationship between Bti interventions and observable changes in egg counts.

## Results

The study period begins with the first Bti treatments on April 30, 2023 (Week 1) and continued through September 27, 2023 (Week 22). During this time, there were 1,320 sampling events representing ovitrap egg counts for 195 unique traps. Each ovitrap was checked on average 6.76 ± 1.13 times. In total 26,653 *Ae. albopictus* eggs were counted during this time period. On average 20.2 ± 52.9 *Ae. albopictus* eggs were counted per trap per check. The median egg count per trap per check across the period was 0 with a maximum egg count of 569. Total egg counts by week steadily increased from 0 in Week 1 and peaked on Week 15 at a total of 5,021. In total there were 4,387 Bti applications during the study period. Bti applications increased steadily until Week 16 where they peaked with 936 applications, then dropping to 8 applications by Week 22. Per trap, the median Bti application was 0 for the whole study period, while the mean Bti application was 3.3 ± 13.8 and the max 138 (Table 1).

**Table 1:** Descriptive statistics of trap-day observations (n = 1320). Variables are summarized at the daily-trap scale, with the exception of vegetation which is time-invariant.

Variable	Mean (SD)	Median	Min–Max
Egg counts per trap-day	20.2 (52.9)	0	0–569.0
Bti applications	3.3 (13.8)	0	0–138.0
Mean daily temperature (°C)	21.1 (2.6)	21.0	15.6–26.7
Daily precipitation (mm)	1.5 (2.8)	0.1	0–23.0
Sparse vegetation (%)	52.5 (3.5)	53.0	44.0–62.0
Dense vegetation (%)	15.6 (10.9)	12.0	0–47.0

## Effects of Bti

**Fig. 1:**
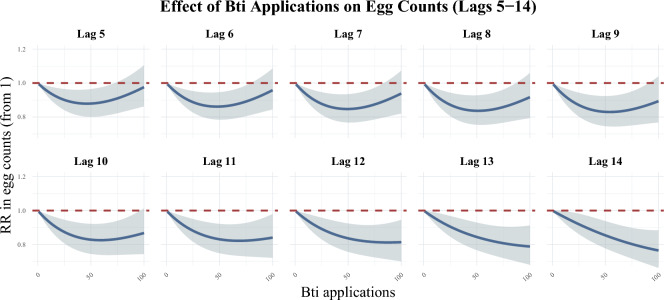
Marginal effects of Bti applications on *Ae. albopictus* egg counts. Values below one represent a relative reduction in eggs. Lags refer to the number of days after a treatment occurred.

At 25 Bti applications, just slightly below the median non-zero Bti application of 27 treatments, statistically significant reductions in *Ae. albopictus* egg counts were observed between Lags 4 and 15 or 4 to 15 days after Bti applications. The associated reduction in egg counts was strongest 6 to 13 days after treatment, where relative rates ranged from 0.91 (95% CI 0.85 – 0.97) to 0.87 (95% CI 0.80 – 0.94), corresponding to approximately 9 – 13% marginal reductions in egg counts. The maximum estimated reduction at 25 Bti treatments was observed 9 days after treatment (RR = 0.87, 95% CI 0.80 – 0.94), equivalent to a 13% marginal reduction in egg counts. The effect of Bti on egg counts begins to diminish in confidence after 15 days for lower treatment levels, though at higher treatment levels the effect remains present up until day 28. For the full results see Fig. S1 (Appendix p.12; Fig. 1).

**Fig. 2:**
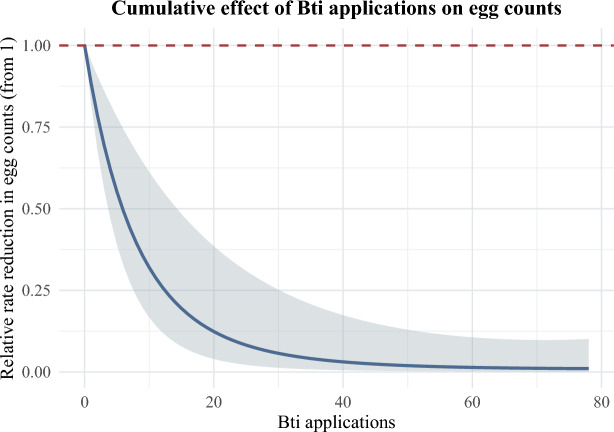
Cumulative effect of Bti applications on *Ae. albopictus* egg counts. Values below 1 represent a relative reduction in eggs.

As seen in Fig. 2, after 28 days the cumulative effect of Bti applications on *Ae. albopictus* egg counts shows a sharp non-linear decline, with the rate of reduction decreasing in magnitude around 20 applications, where the relative reduction in *Ae. albopictus* egg counts approaches 87.4% (95% CI 61.5% – 96.5%). Thereafter the effects of additional Bti treatments diminish with the model estimating a 97% reduction in egg count by 40 treatments (95% CI 82.7% – 99.5%). This suggests a strong maximal efficacy of Bti at even moderate application levels, under field conditions. However, in practice there were relatively few occasions where that many Bti applications occurred, with just 6.0% of ovitraps having 20 or more breeding sites treated within 200 m of them. Though, of the ovitraps with more than 0 Bti applications within 200 m, 63.7% of them did have 20 or more Bti applications.

## Counterfactual scenario

**Fig. 3:**
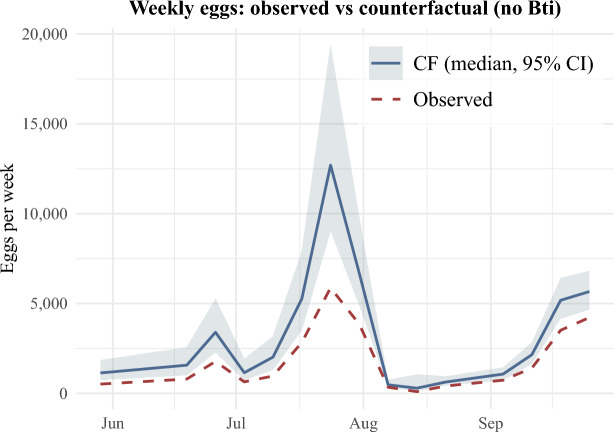
Observed egg counts versus predicted egg counts for the no Bti counterfactual scenario.

Across the entire season, the model estimated 19,201 eggs were prevented by Bti applications (95% CI 8,617 – 37,511), with a peak weekly reduction of 6,451 eggs (95% CI 2,699 – 13,026). Taken together with the 26,653 eggs observed during this study, this implies that, in the absence of control, total egg production would have been 45,854 eggs (95% CI 35,270 – 64,164). In relative terms, Bti treatments implemented during the study reduced total *Ae. albopictus* egg counts by an estimated 41.9% (95% CI 24.4% – 58.5%), with a peak weekly reduction of 65.9% (95% CI 35.5% – 83.6%) and an average reduction of 39.2% (95% CI 22.1% – 51.1%). Bti treatments substantially limited *Ae. albopictus* population growth, especially in the Pfaffengrund cluster where Bti treatments were estimated to have prevented for some individual ovitraps over 1,000 eggs each across the entire season. In the two other clusters, Rohrbach and Kirchheim, Bti treatments were also seen to have significantly prevented additional *Ae. albopictus* eggs, albeit at lower magnitudes. Importantly, the counterfactual scenario suggests that, in the absence of Bti treatments, *Ae. albopictus* eggs would have been observed at least once in all 195 ovitraps across the three neighborhoods, compared to 70.8% of ovitraps in reality (Fig. 3).

## Model performance

**Fig. 4:**
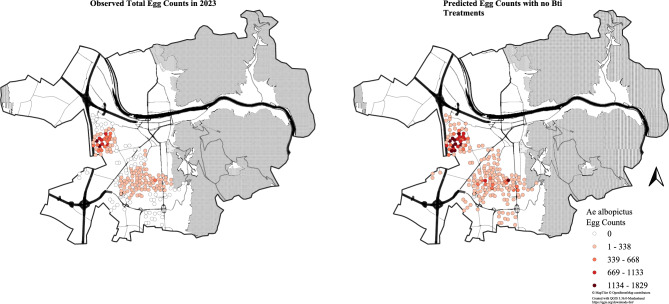
Observed egg counts per ovitrap versus predicted egg counts per ovitrap in the counterfactual scenario.

Overall, the final quasi-Poisson GAM-DLNM model showed good fit. The explained deviance was 69.8% and the adjusted R^2^ was 57.1%. The RMSE was 32.6 and the mean absolute error was 15.6. Additionally, the predicted and observed egg counts were largely aligned with a correlation coefficient of r = 0.79. The estimated dispersion parameter was 29.2, justifying the use of a quasi-Poisson family. Residual temporal autocorrelation was weak, with only a small positive autocorrelation with the first lag (ACF = 0.17) and no persistent temporal structure across later lags, supporting the use of a GAM framework without an explicit temporal correlation component (Fig. 4).

## Spatial effects and covariate effects

A spatial effect was present in the data with three main hotspots representing elevated *Ae. albopictus* egg counts. The three hotspots were present in the urban cores of the neighborhoods Pfaffengrund, Kirchheim, and Rohrbach. Among the three neighborhoods, Pfaffengrund had by far the strongest spatial effect. Peripheral areas for the three neighborhoods were associated with lower egg counts.

Weather covariates had significant associations with *Ae. albopictus* egg counts. In both cases, mean temperature and daily precipitation had highly non-linear effects on egg abundance. Egg counts increased with precipitation up until around 10 mm of precipitation, after which the effect plateaued. Temperature had a much more non-linear relationship with *Ae. albopictus* egg counts, with predicted egg counts generally increasing as temperatures rose above 20 °C. In contrast, after accounting for spatial structures and trap-level differences, local vegetation cover and the monthly seasonal indicators were not statistically significant. Nonetheless, we retained these covariates to adjust for habitat suitability and seasonality, and to reduce potential confounding in the model.

## Discussion

This study provides one of the first field evaluations of the larvicide *Bacillus thuringiensis subsp. israelensis* (Bti) for control of *Ae. albopictus* using a DLNM framework. We found that Bti applications had a clear but time-limited effect on ovitrap egg counts, with the strongest reductions occurring 6 to 13 days after application after which efficacy diminished. These results align with other field studies which found similar efficacy periods for Bti as a control measure for *Ae. albopictus* mosquitoes and other Aedes species.^16–20^ We found that Bti was effective even at moderate treatment counts, though these effects faded sooner over time compared to higher treatment levels which persisted up to 28 days.

Broadly speaking, our findings match previous research on Bti efficacy for *Ae. albopictus* control in Switzerland as well as studies on the efficacy of Bti on the control of *C. pipiens* populations in Italy and *A. darlingi* populations in Brazil.^16,21,22^ Additionally, an experimental study on the catch-basin level testing the efficacy of a larvicide combining both Bti and *Lysinibacillus sphaericus* found that this combined larvicide reduced larvae counts by 100% for up to 28 days with efficacy rates varying between 80–100% reduction up to 70 days post-treatment.^23^ Similarly, another study found that a commercial larvicide containing both Bti and *B. sphaericus* were able to reduce mosquito larvae populations by > 96% in septic tanks for up to 24 days, with substantial residual efficacy up to 31 days.^24^ However, one major difference between these studies and our work is that they analyzed larval presence directly in treated habitats, rather than indirectly through egg counts as we did. This difference highlights the challenge of translating larval suppression in known or controlled breeding sites into population-level reductions in operational settings, where cryptic breeding sites, mosquito dispersal, and the fiscal and logistical constraints of adult surveillance limit the ability to detect reductions in mosquito populations.

The timing of the observed peak efficacy of Bti in our study additionally matches the expected developmental timeline of *Ae. albopictus*. Heidelberg is relatively warm climatically, averaging 21.1 °C across the study period, as such the development time of *Ae. albopictus* will be relatively quick and approximately 7 to 10 days, with an additional 2 to 4 days for adult females to blood-feed and oviposit.^25,26^ As such, Bti as a larvicide would be expected to eliminate mosquito larvae that are both at the tail-end of their development cycle and soon to pupate as well as those recently hatched from eggs. Thus, reductions in ovipositing adult females, and in turn egg counts, would likely be observable within a few days if late-stage larvae are eliminated, with larger reductions occurring over time after a full developmental cycle. Consistent with this timeline, statistically significant reductions in egg counts occurred as early as four days after treatment and continued up to 15 days after treatment. At 25 Bti applications, approximately the median non-zero treatment intensity, reductions ranged from a low of 7% at 15 days (RR = 0.93, 95% CI 0.87 – 0.998) to a maximum reduction of 13% at 9 days (RR = 0.87, 95% CI 0.80 – 0.94).

From a methodological standpoint, the corroboration of our results using DLNM compared with other studies regarding the efficacy of Bti as a vector control for *Ae. albopictus* is heartening. DLNMs offer a powerful framework in which we can estimate both the marginal effects of specific lags as well as the overall non-linear and delayed impacts of variables on an outcome or in this case the impacts of Bti on *Ae. albopictus* egg counts. We were able to apply this framework at a fine spatial scale with ovitraps deployed in a 250 m by 250 m grid across Heidelberg, yielding 1,320 ovitrap checks at 195 unique traps. This hyperlocal sampling allowed us to identify neighborhood-level hotspots at the neighborhood block scale, rather than relying on city or even neighborhood averages. An additional strength of this study is that we were able to translate model outputs into an explicit measure of public health impact by estimating not just relative reductions in egg counts but the absolute number of prevented *Ae. albopictus* eggs for each ovitrap.

Several limitations in this study should be noted. First, we measure *Ae. albopictus* abundance through ovitrap egg counts rather than adult capture. Ovitraps represent the presence of ovipositing females and a relative density of mosquito populations, rather than a true presence or absence of adult mosquitoes. In this study, ovitrap egg counts were used because they provided a cost-effective and operationally feasible means for monitoring *Ae. albopictus* at fine spatial and temporal resolution across an entire season and over a large area. Egg count collection methods such as with ovitraps are also the most commonly used method for large-scale monitoring of *Ae. albopictus*, making our results directly comparable to existing vector monitoring research.^27^ As well, Bti affects larvae rather than adults which means egg counts indirectly capture the effect of Bti due to the time lag between larval mortality and a detectable reduction in egg counts. Moreover, it is important to note that ovitrap egg counts are not a direct measure of reductions in adult mosquito abundance or disease transmission risk. Ovitrap egg counts reflect oviposition behavior and can be influenced by environmental suitability, climatic conditions, as well as other factors, and changes in these egg counts while a good proxy, do not fully capture the population dynamics of adult mosquitoes.^27^.

Second, Bti treatments were not randomized. Vector control efforts in Heidelberg were targeted at areas with known *Ae. albopictus* populations. This results in potential residual confounding, as neighborhoods receiving more intensive Bti applications may have had higher mosquito abundance to begin with due to greater environmental suitability or other factors. As a result, this could bias effect estimates if not accounted for by measured covariates and controls. Similarly, due to unaccounted for factors, an area may have had higher egg counts which then naturally declined over time, regressing to the mean independent of vector control. Without randomization, such declines may be attributed to Bti. We attempted to mitigate these challenges by including trap-level random effects to account for differences between locations, in addition to including a tensor spatial term to capture geographic clustering. A seasonal term was also included to capture temporal trends, as well as climatic covariates and green space terms to adjust for weather and environmental suitability. Nevertheless, confounders may persist, particularly given that we found clear evidence of spatial clustering in *Ae. albopictus* egg counts in three neighborhoods of the city.

Third, the generalizability of these findings relative to other contexts should also be considered. Heidelberg is a relatively warm, central European city with an established and well-funded municipal vector control program. The city is typified by mid-rise and medium density residential areas with substantial green space. These climatic and environmental conditions influence the development rates of *Ae. albopictus*, the availability of breeding sites, and in turn the persistence of the species, factors that can all impact the efficacy of Bti. Similarly, the social environment of Heidelberg and participation of the public in mosquito control efforts will impact the feasibility of localized Bti treatments as a vector control method. The door-to-door interventions and coordinated public-space treatments that occurred in Heidelberg may not be replicable in contexts with limited fiscal resources and lower public engagement. Future work is therefore needed to assess the broader validity of these findings in contexts which may differ socially and environmentally from Heidelberg.

Additionally, Bti treatments on private properties included resident education on the identification and removal of potential breeding sites. The final removal of these breeding sites was at the discretion of the households and data on these removals was not recorded, however. Consequently, the estimated effects attributed to Bti treatments may incidentally reflect the combined impact of Bti treatments as well as unmeasured breeding site removal and behavioral changes in the human populace, rather than the direct effect of Bti on mosquito larvae. At the same time, vector control for *Ae. albopictus* has been ongoing for several years with the mosquito present for nearly a decade. Considering this, many households may have already adopted preventative behaviors prior to this study. As a result, the impact of the educational outreach during the 2023 study period may have been limited, though this cannot be assessed with our data.

Operationally, these findings underscore that containing the spread of *Ae. albopictus* requires persistent and repeated vector control to maintain a high-degree of effect. This is due to the limited efficacy period of Bti, 1–2 weeks in the case of this study, a duration that matches findings in a previous study in Italy.^28^ This limited efficacy period makes it imperative that Bti applications occur in the weeks following favorable weather conditions for *Ae. albopictus* ovipositioning in order to prevent its proliferation. In the case of Heidelberg, the *Ae. albopictus* season stretches from mid-April up until October, but in the context of a warming climate and improving climatic suitability this period is likely to grow. Maintaining this level of effort is challenging socially and logistically, though. Previous research has found that larval source management, which this study assesses, is substantially more expensive than wide-area application techniques such as misting.^29^ However, research in Germany^30^ as well as other countries^31^ has found that public acceptance of Bti as a larvicide varies with large portions of the public uncomfortable or against its use. This suggests that there would likely be even greater public discontent with the implementation of wide-area applications of Bti which instead targeting breeding sites directly, disperse Bti across large swaths of land indiscriminately.

Our results suggest that hyperlocalized Bti treatments may be capable of preventing systemic establishment of *Ae. albopictus,* which is a key step in reducing the likelihood of vector spillover from one community to another. Such prevention is paramount, especially from the climate adaptation perspective, as the local establishment of *Ae. albopictus* has been shown to often be followed by outbreaks of Dengue and Chikungunya.^6^ Further, reducing mosquito abundance, particularly during peak season, can reduce outbreak potential by lowering the basic reproduction number.

Moreover, our results suggest that systemic establishment may be preventable without ubiquitous Bti treatment. This implies that municipal vector control programs can spatially optimize their control efforts to target high impact areas, resulting in lower costs and fewer externalities including public discontent with vector control. It is also evidence that if we are to limit the spread of *Ae. albopictus*, one viable option is to upscale vector control programs like Heidelberg’s to other municipalities as the program was not only highly effective, but of a form that is more likely to garner public acceptance. However, we also saw that this still took significant community collaboration as these treatments largely took place on residential properties. Participation fatigue is therefore a significant potential barrier. Suggesting that as *Ae. albopictus* becomes fully established in Europe, like it is in Heidelberg, this may lead to a situation where for mosquito control efforts to be successful, it cannot just be a question of improving eradication technology or methods, but also of community engagement, participation, and buy-in.

## Materials and methods

### Study area and period

**Fig. 5:**
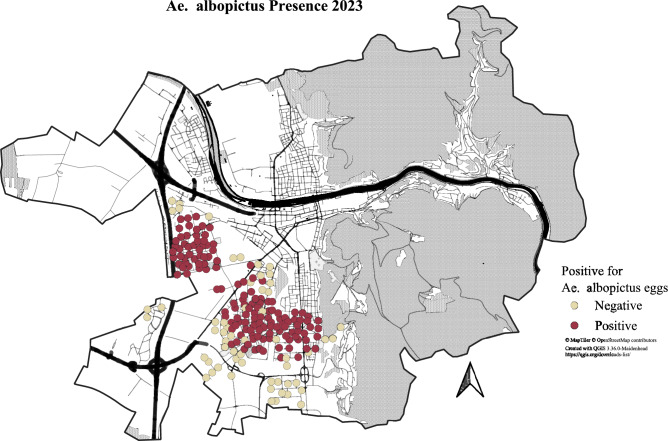
*Ae. albopictus* presence positive or negative per ovitrap for the 2023 season. Traps positive for *Ae. albopictus* at any point in the season are marked red.

The study area consists of the city of Heidelberg (49.41101° N, 8.68286° E), Germany and sits at the northeastern end of the Upper Rhine Valley around one hundred kilometers from the French border. Climatically, Heidelberg is characterized by wet winters and hot summers and it is situated in the warmest region of Germany^32^. Heidelberg has a population of around 160,000 with an urban core typified by mid-rise buildings with scattered suburban clusters (Fig. 5).

*Ae. albopictus* was first detected in Heidelberg in 2016. Since then, municipal authorities have employed mosquito control experts to reduce *Ae. albopictus* populations using measures including the sterile insect technique, Bti, and community outreach to promote social behavior that can reduce mosquito populations such as the removal of potential breeding sites in private residences. This study focuses on the period from April 2023 to October 2023. During this time, Bti and community outreach were the only vector control methods practiced.

## Mosquito egg surveillance

In order to monitor *Ae. albopictus* populations in Heidelberg, the city in partnership with the local mosquito control group maintains a network of 544 ovitraps deployed across the city in a 250 m x 250 m grid with a minimum of 1 ovitrap per grid cell. This allows for the complete surveillance of *Ae. albopictus* throughout the city. *Ae. albopictus* is highly clustered in Heidelberg with three clusters present in the neighborhoods of Kirchheim, Rohrbach, and Pfaffengrund. These clusters have persisted despite significant Bti application as well as household visits by the local mosquito control group where they advised residents on potential breeding sites for the mosquitos and the removal of these breeding sites. For the purposes of this study, we have centered our research on the 195 ovitraps in these neighborhoods as they were the only areas of Heidelberg to have persistent *Ae. albopictus* populations and thus were most suited for measuring the efficacy of Bti. These ovitraps were sampled every two to three weeks.

## Bti application protocol

Control measures targeting *Ae. albopictus* were implemented across Heidelberg based on entomological surveillance data from 2022 and updated throughout the 2023 season. Intervention rounds were carried out between April and October 2023 by trained professionals, each consisting of coordinated Bti treatments in public spaces and door-to-door activities on private properties. Public-space treatments involved the application of the biological larvicide VectoBac® WG (containing *Bacillus thuringiensis subsp. israelensis*, strain AM65-52, 3000 ITU/mg) to all identified mosquito breeding sites in accessible outdoor environments. The larvicide was applied using handheld equipment, with a standardized dosage of 83.3 g of VectoBac® WG per liter of water. Door-to-door interventions on private properties consisted of three standardized components: (1) resident education on the biology, public health relevance, and control of *Ae. albopictus*, (2) identification and assessment of breeding sites on the property, and (3) Bti treatment of all identified sites using VectoBac® WG. Both public space and door-to-door interventions were repeated every two-weeks from April to October. During door-to-door visits, residents were advised on the identification and removal of potential breeding sites. However, the removal of these sites was performed at the discretion of the household and was not recorded. The intervention metric in this study therefore is the number of treated breeding sites within 200 m of each ovitrap and does not directly capture the potential impact of the removal of breeding sites from private properties.

For each Bti application—whether in public spaces or as part of door-to-door interventions—the date and geographic coordinates of the treatment location were recorded using handheld GPS-enabled devices. Bti applications were carried out on any potential breeding sites identified by the mosquito control personnel, including artificial breeding containers such as flower pots or watering cans and catch basins. As Bti interventions were repeated across the season, each individual Bti application represents the treatment of a single breeding site at that given time. As such, if the same breeding site was present during the next round of interventions it would receive an additional Bti application at that time. In line with the processing of other environmental variables, the Bti treatment data were spatially joined to each ovitrap location using a 200-m radial buffer. The resulting dataset was then transformed into a daily time series, capturing the number of Bti applications conducted within the 200-m buffer zone surrounding each ovitrap for every day of the study period.

## Environmental data

Environmental data in this study includes Sentinel-2 satellite imagery for NDVI, stationary weather sensors and mobile-bike mounted sensors for mean daily temperature, and daily precipitation from the German Weather Service.^33,34^ For NDVI, we split it into two categories: sparse green (NDVI 0.2—0.6) and dense green spaces (NDVI > 0.6). These NDVI thresholds were selected based on recommendations by the United States Geological Survey.^35^ We modeled air temperature using a spatial kriging model based on hourly temperature residuals and elevation. Subsequently, 200-m radial buffer zones were created around each ovitrap to extract localized data for the above environmental variables, thereby providing an estimate of the microclimatic conditions surrounding each trap. The size of the buffer zone was selected based on previous research on the flight range *Ae. albopictus* mosquitoes.^36^.

## Modeling approach

A distributed lag non-linear model (DLNM) was developed to assess the lagged effects of Bti on *Ae. albopictus* egg counts.^37^ The model was fitted as generalized additive models (GAM) using a quasi-Poisson distribution and the restricted estimation maximum likelihood (REML) method was used for parameter estimation.^38^ The outcome was a count of *Ae. albopictus* eggs per trap. A quasi-Poisson distribution was selected to enable relative-risk estimations for the effect of Bti treatments on *Ae. albopictus* egg counts. A GAM approach was taken as an alternative to a generalized additive mixed model (GAMM), as a GAM approach facilitates the joint estimation of DLNM terms with other smoothed covariates.

Although ovitraps were inspected every two to three weeks, the data for this analysis were constructed as a daily time series for analysis. Egg counts were assigned to their respective sampling date for each trap with the days between assigned as non-observation days, while Bti exposure was operationalized as the daily Bti treatment counts applied within a 200-m buffer surrounding each ovitrap. Bti treatments therefore represent treatment intensity on a per trap-level, rather than a binary treated or non-treated classification. The analysis was done at the daily level in order to align the ovitrap data with the daily Bti application data as well to facilitate the modeling of the lagged treatment effects from Bti.

The cross-basis function for the lagged effects of Bti on *Ae. albopictus* egg counts assumed a b-spline for the exposure–response relationship with three degrees of freedom and a natural cubic spline structure for the lag-response relationship with four degrees of freedom. The b-spline structure for the exposure–response relationship additionally included boundary knots at 0 and 100 Bti treatments, representing 0 to 99 percentile of daily Bti treatments per ovitrap. The lag period assessed ranged from the day of treatment to 28 days after treatment. In interpretation, this means that the individual lags, e.g. Lag 4, correspond to the ongoing efficacy of Bti 4 days after the treatment application occurred. The 28-day lag period was selected based on previous research which found that the effective duration of Bti for controlling *Ae. albopictus* ranged from one week to up to four weeks in field settings^17,39^. This variability motivated the use of a 28-day lag window in order to assess both the short-term and potential extended effects of Bti.

The spline specifications for both the exposure–response and lag–response dimensions of the model were chosen to provide sufficient flexibility to capture potential non-linear effects. Sensitivity analyses were conducted to evaluate alternative lag structures for the DLNM model and to assess model robustness using *Ae. albopictus* presence/absence instead of egg counts. Across the alternative lag structures, the overall pattern of Bti effectiveness was consistent. Although a less flexible cross-basis achieved a slightly lower quasi-AIC than the original model, both models produced highly similar effect estimates for Bti across the exposure range. We therefore retained the original model to maintain interpretability across models. The binomial (presence/absence) sensitivity analysis similarly demonstrated that Bti treatments reduced the likelihood of finding *Ae. albopictus* eggs, with the strongest effects observed one- to two-weeks post-treatment.

Additional covariates in the model include a spatial interaction term that was modeled as a tensor product of thin-plate regressions for latitude and longitude. A random intercept per trap ID was included as a penalized smooth term to account for repeated trap sampling over time and persistent differences between traps. A month term was included to account for seasonality. Dense and sparse green space were also included as controls. Mean daily temperature and daily precipitation were included as smoothed terms to allow for flexible, non-linear associations between climatic conditions and egg counts. Precipitation was included only for the same day as the sampling. This was decided after performing a sensitivity analysis comparing the inclusion of the smoothed same-day precipitation term versus 7-day, 14-day, and 21-day cumulative precipitation terms. Of these four options, the model with the same-day precipitation term had the lowest quasi-AIC, suggesting a better balance of model fit and parsimony, although the cumulative precipitation models had slightly higher deviance explained.

In addition to the GAM-DLNM model, we developed a counterfactual scenario where no Bti treatments occurred in Heidelberg. In this scenario, we perform two sets of predictions, one where we predict the number of eggs expected per trap for each sampling period given the actual Bti treatments each trap received during the respective sampling period, and one where we predict egg counts per trap for each sampling period as if those traps had zero Bti treatments occur in their vicinity. The estimated number of eggs prevented on each trap-day was then calculated as the difference between these two predictions for each observation respectively, with the total prevented eggs calculated by summing these differences across all observations. To estimate the uncertainty of these predictions, a Monte Carlo simulation was performed using the estimated coefficient variance–covariance matrix in the fitted GAM. This simulation was repeated 300 times, each time recomputing the predicted egg counts and the total eggs prevented to then obtain a 95% confidence interval.

It bears noting that although the counterfactual scenario does rely on the assumption that aside from the covariates included in the model, there were no other significant unmeasured factors influencing the mosquito population dynamics and the timing and intensity of Bti treatments. Moreover, because Bti interventions targeted areas with known *Ae. albopictus* populations and were not random, the counterfactual scenario should be interpreted as a model projection rather than a causal estimation resulting from a randomized experiment.

All analyses were conducted in R version 4.3.2. All DLNMs were implemented with the dlnm package^40^ and fitted within a generalized additive modeling framework using the mgcv package.^41^.

## Supplementary Information

Supplementary Information.

## Data Availability

The materials and datasets generated and analyzed during this study are available from the corresponding author upon reasonable request. Restrictions apply only to the sharing of entomological surveillance data collected by the ICYBAC Mosquito Control GmbH on behalf of the city of Heidelberg, for which access should be granted directly from there. Meterological data from HYRAS is available at: https://www.dwd.de/DE/leistungen/hyras/hyras.html. Air temperature from city of Heidelberg can be found here: https://ckan.datenplattform.heidelberg.de/de/dataset/environment_main_barani. Green space data was directly sourced from OpenStreetMap using the Ohsome API.

## Code availability

The code to reproduce the models and figures can be found at: https://github.com/cstarob/Temporal-Spatial-Bti-Albopictus.
