# Supplementary material for: Fine-scale temporal and spatial dynamics of Ae. albopictus response to larviciding with Bacillus thuringiensis israelensis in Heidelberg, Germany
Source: Sci Rep. 2026 Apr 8;16:12031. doi: 10.1038/s41598-026-46094-9 (PMC13068888; doi:10.1038/s41598-026-46094-9)
Supplement: Supplementary file 1 — Supplementary Information. [file 41598_2026_46094_MOESM1_ESM.docx]

**Appendix**


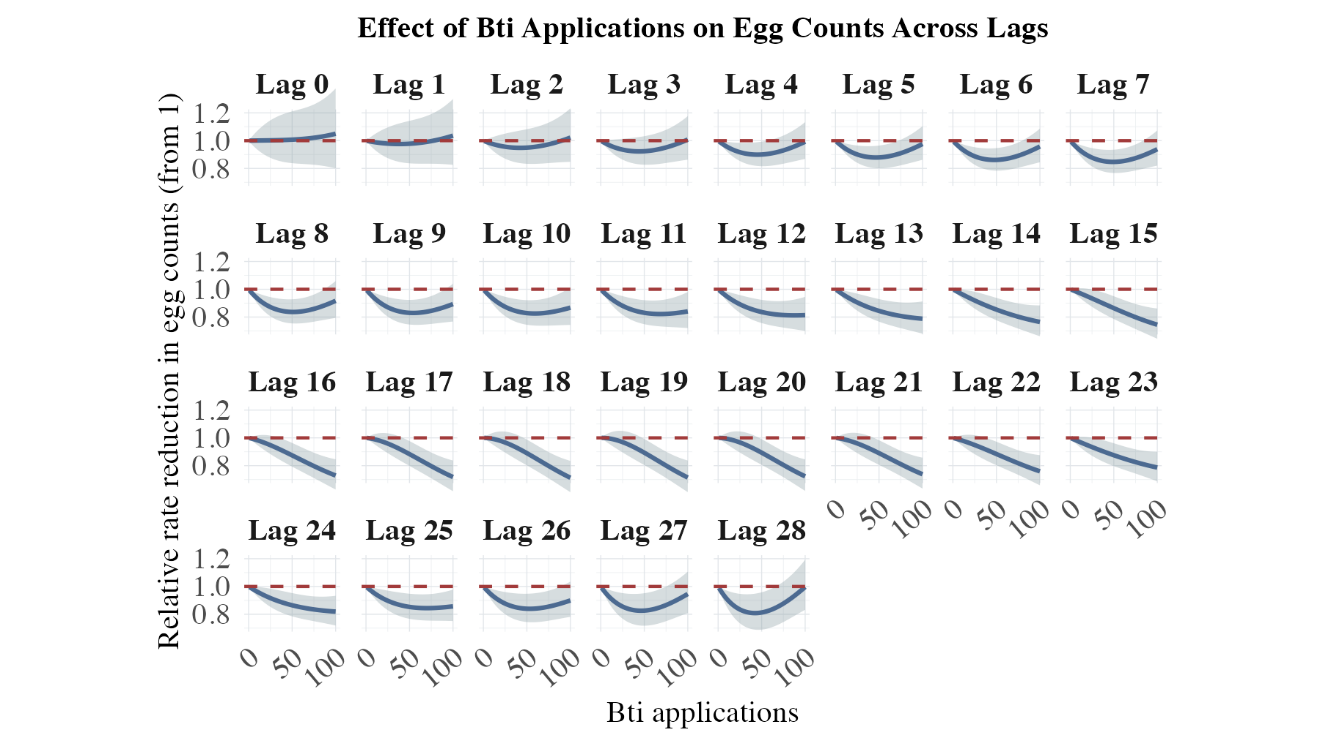


Figure S1 Marginal effects of Bti applications on Ae. albopictus egg counts. Values below one represent a relative reduction in eggs. Lags correspond to days after the treatment occurred.

| **Model** | **AIC / quasi-AIC** | **Deviance Explained** | **Adjusted R^2^** |
| --- | --- | --- | --- |
| Quasi-Poisson 14-day lag (B-spline exposure, 3 df; natural spline lag, 4 df) | 1634.19 | 0.7 | 0.58 |
| Quasi-Poisson 21-day lag (B-spline exposure, 3 df; natural spline lag, 4 df) | 1604.07 | 0.7 | 0.58 |
| Quasi-Poisson 28-day lag (linear exposure–response; natural spline lag, 3 df) | 1583.15 | 0.69 | 0.55 |
| Quasi-Poisson 28-day lag (reduced flexibility: B-spline exposure, 2 df; natural spline lag, 3 df) | 1596.67 | 0.7 | 0.57 |
| Quasi-Poisson 28-day lag (baseline flexibility: B-spline exposure, 3 df; natural spline lag, 4 df) | 1609.87 | 0.7 | 0.57 |
| Quasi-Poisson 28-day lag (increased flexibility: B-spline exposure, 4 df; natural spline lag, 5 df) | 1630.14 | 0.71 | 0.59 |
| Binomial 14-day lag (B-spline exposure, 3 df; natural spline lag, 4 df) | 986.93 | 0.53 | 0.53 |
| Binomial 21-day lag (B-spline exposure, 3 df; natural spline lag, 4 df) | 984.84 | 0.52 | 0.53 |
| Binomial 28-day lag (linear exposure–response; natural spline lag, 3 df) | 1002.45 | 0.50 | 0.51 |
| Binomial 28-day lag (reduced flexibility: B-spline exposure, 2 df; natural spline lag, 3 df) | 1055.84 | 0.49 | 0.50 |
| Binomial 28-day lag (baseline flexibility: B-spline exposure, 3 df; natural spline lag, 4 df) | 997.44 | 0.50 | 0.51 |
| Binomial 28-day lag (increased flexibility: B-spline exposure, 4 df; natural spline lag, 5 df) | 996.49 | 0.51 | 0.52 |
| Same-day precipitation & same-day mean temperature | 1609.87 | 0.7 | 0.57 |
| Same-day precipitation & 7-day mean temperature | 1615.01 | 0.69 | 0.56 |
| Same-day precipitation & 14-day mean temperature | 1615.01 | 0.69 | 0.56 |
| Same-day precipitation & 21-day mean temperature | 1615.01 | 0.69 | 0.56 |
| 7-day cumulative precipitation & same-day mean temperature | 1613.32 | 0.7 | 0.56 |
| 7-day cumulative precipitation & 7-day mean temperature | 1602.56 | 0.7 | 0.57 |
| 7-day cumulative precipitation & 14-day mean temperature | 1602.56 | 0.7 | 0.57 |
| 7-day cumulative precipitation & 21-day mean temperature | 1602.56 | 0.7 | 0.57 |
| 14-day cumulative precipitation & same-day mean temperature | 1629.85 | 0.7 | 0.57 |
| 14-day cumulative precipitation & 7-day mean temperature | 1639.12 | 0.71 | 0.59 |
| 14-day cumulative precipitation & 14-day mean temperature | 1639.12 | 0.71 | 0.59 |
| 14-day cumulative precipitation & 21-day mean temperature | 1639.12 | 0.71 | 0.59 |
| 21-day cumulative precipitation & same-day mean temperature | 1635.37 | 0.71 | 0.59 |
| 21-day cumulative precipitation & 7-day mean temperature | 1687.21 | 0.72 | 0.6 |
| 21-day cumulative precipitation & 14-day mean temperature | 1687.21 | 0.72 | 0.6 |
| 21-day cumulative precipitation & 21-day mean temperature | 1687.21 | 0.72 | 0.6 |
| Bti cross-basis + temperature and precipitation | 733.14 | 0.25 | 0.14 |
| Bti cross-basis + temperature and precipitation + spatial smooth (longitude–latitude interaction) | 1221.31 | 0.49 | 0.35 |
| Bti cross-basis + temperature and precipitation + spatial smooth (longitude–latitude interaction) + trap-level random intercept + month (seasonality) | 1608.3 | 0.7 | 0.57 |
| Bti cross-basis + temperature and precipitation + spatial smooth (longitude–latitude interaction) + trap-level random intercept + month (seasonality), excluding vegetation covariates | 1608.3 | 0.7 | 0.57 |
| Bti cross-basis + temperature and precipitation + spatial smooth (longitude–latitude interaction) + trap-level random intercept + month (seasonality) + sparse and dense vegetation covariates | 1609.87 | 0.7 | 0.57 |
| Bti cross-basis + temperature and precipitation + spatial smooth (longitude–latitude interaction) + trap-level random intercept | 1697.27 | 0.61 | 0.48 |

Table S2 Sensitivity analyses comparing alternative cross-basis specifications, environmental covariates, and lag specifications for climatic features.


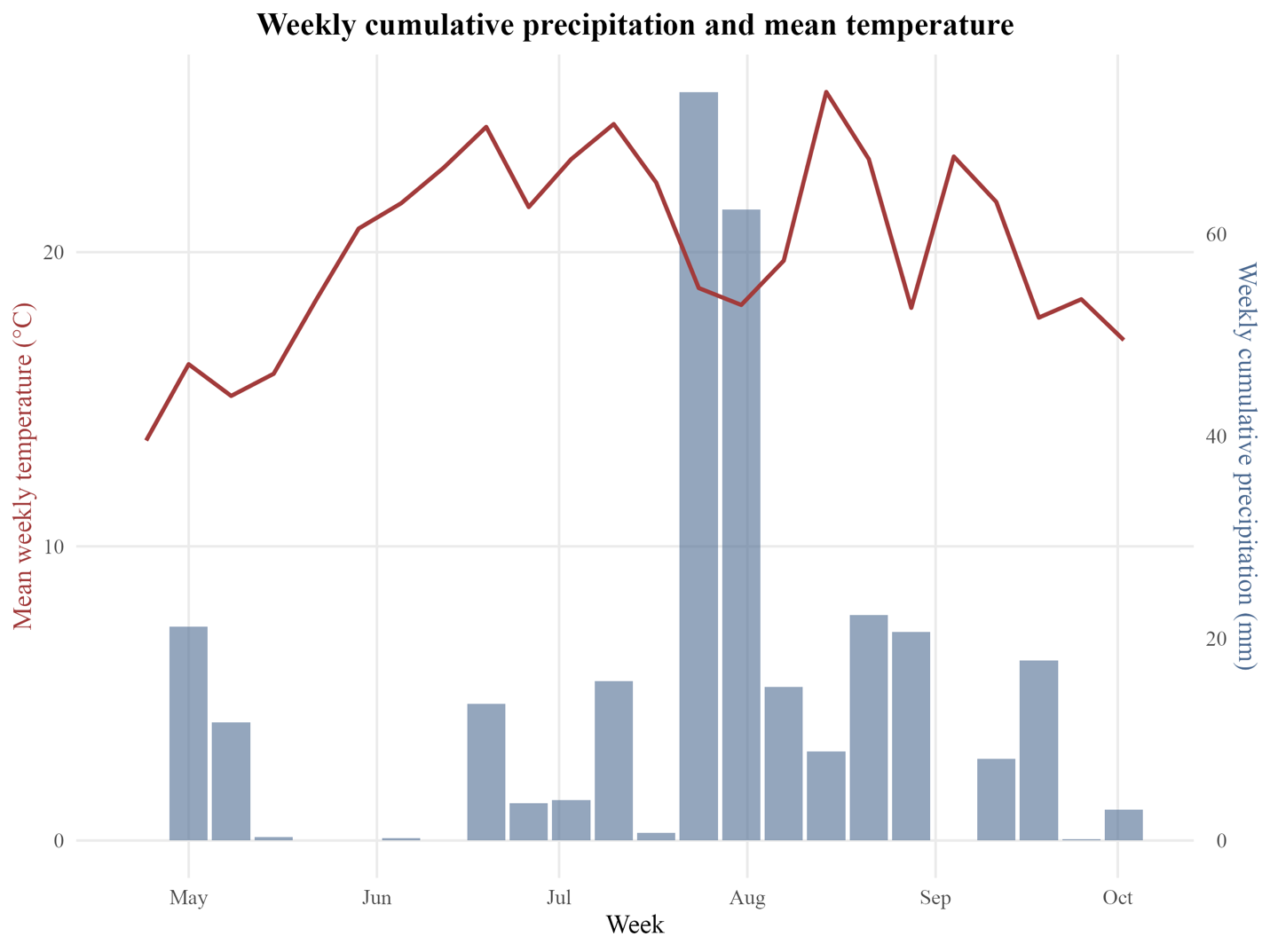


Figure S3 Mean weekly temperature and weekly cumulative precipitation across the entire study period.
